# Silencing of long noncoding RNA MIAT inhibits the viability and proliferation of breast cancer cells by promoting miR-378a-5p expression

**DOI:** 10.1515/med-2023-0676

**Published:** 2023-04-03

**Authors:** Chao Yan, Yue Jin

**Affiliations:** Medical Laboratory, The Affiliated Huai’an Hospital of Xuzhou Medical University and The Second People’s Hospital of Huai’an, Huai’an 223003, Jiangsu, China; Medical Laboratory, The Affiliated Huai’an Hospital of Xuzhou Medical University and The Second People’s Hospital of Huai’an, No. 62, Huaihai South Road, Qingjiangpu District, Huai’an 223003, Jiangsu, China

**Keywords:** breast cancer, MIAT, miR-378a-5p, viability, proliferation

## Abstract

Myocardial infarction–associated transcript (MIAT) is a long noncoding RNA that plays a critical role in a variety of diseases. Accordingly, this study probed into the possible interaction mechanism between MIAT and miR-378a-5p in breast cancer. Concretely, MIAT and miR-378a-5p expressions in breast cancer tissues and cells were measured. After transfection with siMIAT and miR-378a-5p inhibitor, the viability and proliferation of breast cancer cells were examined by cell counting kit-8 and colony formation assays. The expressions of apoptosis-related proteins were detected. According to the results, MIAT was highly expressed in breast cancer tissues and cells. MIAT silencing could decrease Bcl-2 expression, viability, and proliferation of breast cancer cells and increase the expressions of cleaved caspase-3 and Bax. MIAT and miR-378a-5p could directly bind to each other, and MIAT silencing promoted the expression of miR-378a-5p. miR-378a-5p expression was low in breast cancer tissues. The miR-378a-5p inhibitor enhanced the viability and proliferation of breast cancer cells and partially reversed the effects of MIAT silencing on the breast cancer cells. In conclusion, MIAT silencing inhibits the viability and proliferation of breast cancer cells by promoting miR-378a-5p, indicating the potential of MIAT as a new target for the treatment of breast cancer.

## 1 Introduction

Breast cancer is a common malignant tumor that occurs in the glandular epithelial tissues of the breast [1], with the incidence increasing year by year [2]. Owing to the high mortality rate, breast cancer has currently emerged as one of the major diseases that seriously endanger women’s life and health worldwide [2]. The clinical symptoms of patients with breast cancer are insidious at the early stages. However, at the time of diagnosis, the clinical manifestations were quite obvious, and even lymph node metastasis occurs, which greatly affects the prognosis and survival rate of patients [3,4,5,6]. In recent years, with the development of bioinformatics and the in-depth studies of the mechanism of breast cancer, target therapy for breast cancer has become a research hotspot [7].

Long noncoding RNAs (lncRNAs) refer to a type of noncoding RNA with over 200 nucleotides in length and no protein-coding potential [8]. A study has demonstrated that a variety of lncRNAs are involved in cancer progression, and their misregulation and mutations may play important roles in cancer [9]. Myocardial infarction–associated transcript (MIAT) is a lncRNA conserved in assorted species that was first reported in mitotic retinal precursor cells [10]. In recent years, MIAT has been proved to be implicated in the development of diversified human diseases, especially tumors [11]. For instance, MIAT is highly expressed in lung cancer and neuroendocrine prostate cancer and interacts with multiple genes to participate in cancer development, which has been widely perceived as a therapeutic target [12,13]. Likewise, MIAT is highly expressed in breast cancer, and inhibition of MIAT can repress breast cancer cell migration and proliferation and promote apoptosis [14]. In addition, it has been reported that MIAT silencing induces the apoptosis of breast cancer cells and enhances cell sensitivity to chemotherapy drugs [15]. Although the role of MIAT in breast cancer has been partially reported, its regulatory mechanism needs to be further analyzed.

In the past few years, researchers have discovered a new mode of regulation in cancer in which lncRNAs can regulate their expressions by competitively binding to related microRNAs (miRNAs), further affecting the progression of cancer [9,16,17]. For example, lncRNA growth-stasis-specific transcript 5 can promote apoptosis by targeting and regulating the expression of miR-378a-5p in triple-negative breast cancer [18]. In addition, several reports have uncovered that miR-378a-5p is able to regulate the proliferation, angiogenesis, apoptosis, and migration of cancer cells [19,20]. For example, miR-378a-5p may serve as a tumor suppressor gene in colorectal cancer [20], and miR-378a-5p expression has a correlation with the occurrence of breast cancer tumors [21]. The balance between cell apoptosis and proliferation is important in a wide variety of physiological settings. Dysfunctional apoptosis and uncontrolled proliferation can result in assorted diseases, including cancer [22,23]. Cancer is one of the conditions where there is too little apoptosis to cause malignant cells to die [24]. BCL-2 family members play integral roles in apoptosis, among which cleaved caspase-3 and Bax are pro-apoptotic genes and Bcl-2 is an anti-apoptotic gene [25]. Based on the abovementioned information, the effect of miR-378a-5p on the apoptosis of breast cancer cells was further explored in this study.

Herein, we explored the interaction mechanism of miR-378a-5p and MIAT in breast cancer, with the aim of uncovering a new molecular mechanism of breast cancer development and providing novel cues for the treatment of breast cancer.

## 2 Materials and methods

### 2.1 Patient tissue specimens

In this study, 30 breast cancer tissue samples were collected from patients at Huai’an Second People’s Hospital on March 19, 2022. All patients were pathologically diagnosed with breast cancer and had not received preoperative treatment. At the same time, normal tissues around the breast cancer tissue were collected as the control group. The clinical data of all patients are shown in Table 1, including age, estrogen receptor (ER) status, tumor size, tumor grades, low grade, high grade, progesterone receptor (PR) status, P53 status, tumor stage, subtype, histology, and human epidermal growth factor 2 (HER2) status.

**Table 1:** The clinical data of all breast cancer patients in this study

Characteristics	Numbers (%)	Mean of 2^△ct^ ± SE	*P*-value MIAT
Age			0.017*
<45 years	11 (36.7)	0.032 ± 0.012	
≥45 years	19 (63.3)	0.017 ± 0.005	
Tumor size (V/cm^3)^			0.028*
<14 cm^3^	17 (56.7)	0.015 ± 0.037	
≥14 cm^3^	13 (43.0)	0.036 ± 0.009	
Tumor grades			0.071
Ⅰ	8 (26.7)	0.018 ± 0.003	
Ⅱ	7 (23.3)	0.022 ± 0.007	
Ⅲ	10 (33.3)	0.029 ± 0.013	
Ⅳ	5 (16.7)	0.029 ± 0.005	
Low grade	17 (56.7)	0.018 ± 0.007	0.003**
High grade	13 (43.3)	0.031 ± 0.015	
ER status			0.023*
Negative	5 (16.7)	0.017 ± 0.056	
Positive	25 (83.3)	0.026 ± 0.008	
PR status			0.360
Negative	11 (30.0)	0.018 ± 0.053	
Positive	19 (70)	0.028 ± 0.009	
HER2 status			0.020*
Negative	9 (23.3)	0.020 ± 0.004	
Positive	21 (76.7)	0.036 ± 0.019	
P53 status			<0.001***
Negative	19 (63.3)	0.032 ± 0.013	
Positive	11 (36.7)	0.009 ± 0.003	
Stage			0.046*
Ⅰ–Ⅱ	21 (70.0)	0.031 ± 0.014	
Ⅲ–Ⅳ	9 (30.0)	0.041 ± 0.015	
Subtype			0.057
Luminal A	16 (53.3)	0.049 ± 0.020	
Luminal B	5 (16.7)	0.042 ± 0.039	
Triple negative	3 (10.0)	0.016 ± 0.012	
HER2 Tyre	2 (6.0)	0.008 ± 0.003	
Unclassified	4 (13.3)	0.036 ± 0.017	
Histology			0.091
Lobular	5 (16.7)	0.097 ± 0.142	
Ductal	25 (83.3)	0.097 ± 0.058	

*P < 0.05, **P < 0.01, ***P < 0.001.

### 2.2 Cell culture

Five cell lines purchased from ATCC (MD, USA) were selected for this experiment, including one normal breast epithelial cell line, MCF-10A (CRL-10317), and four breast cancer cell lines, MDA-MB-231 (HTB-26), SK-BR-3 (HTB-30), BT-20 (HTB-19), and MDA-MB-436 (HTB-130). MCF-10A cells were cultured in the MEBM (CC-3151; Lonza, Basel, Switzerland), containing the components from the Medium Kit (CC-3150; Lonza, Basel, Switzerland). MDA-MB-231 cells were cultured in the specific medium (CM-0150; Procell, Wuhan, China); SK-BR-3 cells were cultured in the specific culture medium (CM-0211; Procell); BT-20 cells were cultured in the specific culture medium (CM-0324; Procell); and MDA-MB-436 cells were cultured in their specific culture medium (CM-0383; Procell).

### 2.3 Cell transfection

After the SK-BR-3 and MDA-MB-231 cell lines were collected, the cell concentration was adjusted. Then, cells were transferred into six-well plates in two parts and cultured for later transfection experiments. In the first part, small interfering RNAs targeting MIAT (siMIAT; target sequence: 5′-GAGGCTTTACAGCCTGTAATTCT-3′) and the negative control of siMIAT (siNC; 5′-CAAATCACAGAATCGTCGTAT-3′) were severally transfected into cells. In the second part, siNC/siMIAT and miR-378a-5p inhibitor (I; 5′-ACACAGGACCUGGAGUCAGGAG-3′)/inhibitor control (IC; 5′-CAGUACUUUUGUGUAGUACAA-3′) were co-transfected into SK-BR-3 and MDA-MB-231 cell lines. When cells reached 80% confluence, the transfection was performed as indicated in the Transfection Reagent Kit (L3000150; Thermo Fisher, MA, USA). All the above cell lines were transfected for 48 h.

### 2.4 Dual-luciferase reporter assay

The wild-type sequence (WT; 5′-AAACCUGGCAGAUGGUCCUAGGUCAGGAU-3′) and mutant sequence (MUT; 5′-AAACCUGCUCGGUAAUGACAUCAGGCAGU-3′) of MIAT specifically synthesized in combination with miR-378a-5p were inserted into the dual-luciferase reporter vector pmirGLO (Promega, Madison, WI, USA) to construct dual-luciferase reporter plasmids (pmirGLO-MIAT-WT and pmirGLO-MIAT-MUT). In this experiment, SK-BR-3 and MDA-MB-231 cells (5 × 10^5^ cells/well) were cultured in the six-well plate. About 5 µg pmirGLO-MIAT-WT or pmirGLO-MIAT-MUT, 100 nM mimic control (Blank) or miR-378a-5p mimic, and Lipofectamine 3000 reagent were diluted with Opti-MEM medium, respectively. Then, the dilutions were mixed and maintained at room temperature for 10 min. After that, these lipid complexes were added to incubate the SK-BR-3 and MDA-MB-231 cells at 37℃ for 48 h. The activity of luciferase was tested on the dual-luciferase reporter system (30IOC; Promega) using a dual-luciferase assay kit (D0010; Solarbio, Beijing, China).

### 2.5 Cell counting kit-8 (CCK-8) assay

The viability of SK-BR-3 and MDA-MB-231 cells after transfection was measured using the CCK-8 Cell Viability Assay Kit (KGA317; Keygen, Nanjing, China). Briefly, cells (3 × 10^3^) were collected and cultured in 96-well plates according to the instructions. After the cells were treated and cultured continuously for 48 h, and 10 µl CCK-8 reaction solution was added to the well. Next, the cells were cultured for another 2 h, and then the absorbance of cells in each well was measured at 450 nm by a microplate reader (EnSight; PerkinElmer, MA, USA).

### 2.6 Colony formation assay

SK-BR-3 and MDA-MB-231 cells in each group were separately collected 48 h after transfection, washed with phosphate buffer saline (PBS; C0221A; Beyotime, Shanghai, China), and later centrifuged at 1,000 × *g* for 5 min on a centrifuge (HT175R; Cence, Changsha, China). Thereafter, the number of cells was counted and then added to 6-well plates (800 cells/well). Colony formation was observed after the cells were cultured for ∼2 weeks. Then, cells were rinsed with PBS and treated with 4% paraformaldehyde (158127; Sigma–Aldrich, Missouri, USA) for 10 min. Later, Giemsa working solution (C0131; Beyotime) was used to stain the cells for 10 min, followed by PBS washing. The number of colony formations per well was observed and recorded under an inverted microscope (XDS-1B; Liuhui Science, Chongqing, China). Colonies containing more than 50 cells were counted.

### 2.7 Quantitative real-time polymerase chain reaction (qRT-PCR)

The transfected cells and tissues were collected in centrifuge tubes. To be specific, the cells were washed with PBS and the collected tissues were homogenized. Later, the total RNA was extracted using the Total RNA Isolation Kit (AM1914; Thermo Fisher) and miRNAs were extracted using the miRNA Isolation Kit (K157001; Thermo Fisher). After that, RNA purity and concentration were analyzed by the agarose gel electrophoresis and spectrophotometer (Evolution 350; Thermo Fisher), respectively. The reverse transcription was implemented as per the instructions of the cDNA Reverse-transcription Kit (D7170S; Beyotime), and the cDNA was amplified in the PCR instrument. The expressions of genes were detected on the RT-PCR system (ABI 7500; Applied Biosystems, CA, USA) with the PowerUp™ SYBR™ Green Master Mix (A25742; Thermo Fisher). All primer sequences are listed in Table 2. Glyceraldehyde-3-phosphate dehydrogenase (GAPDH) and U6 acted as the reference genes. The data were analyzed by the 2^−ΔΔct^ method.

**Table 2:** All primers in RT-PCR experiments in this study

ID	Forward sequence (5′-3′)	Reverse sequence (5′-3′)
MIAT	TCTTCATGTCAGAACACGCTTTA	AAGGTCACCCGAGGTCCAA
miR-378a-5p	CAAACCTCCTCCTGACTCCAG	TATGCTTGTTCTCGTCTCTGTGTC
U6	CTCGCTTCGGCAGCACA	ACGCTTCACGAATTTGCGT
GAPDH	CAATGACCCCTTCATTGACC	GACAAGCTTCCCGTTCTCAG

### 2.8 Western blot

The treated cells from each group were collected and transferred into a centrifuge tube. Then, the appropriate amount of lysis buffer (R0030; Solarbio) was added to the centrifuge tube, and the total proteins were extracted from the cells. The protein standard sample and bicinchoninic acid (BCA) working solution were prepared according to the specification of the BCA Protein Assay Kit (P0012S ). Next, the protein concentration was determined. The prepared gel (AR0138; BOSTER, Wuhan, China) was installed into the electrophoresis tank, after which the protein sample to be tested and the protein marker were added into the gel well for the electrophoresis experiment. Afterward, the proteins were transferred to a polyvinylidene difluoride (PVDF) membrane (YA1701; Solarbio). The PVDF membrane was soaked in the prepared 5% skimmed milk for 2 h and rinsed three times with Tris-buffered saline with Tween 20 (TBST; Solarbio) for 5 min. The PVDF membrane was then incubated with primary antibodies at 4℃ overnight, washed with TBST, and cultured with secondary antibodies at room temperature for 1.5 h. Later, the membrane was completely immersed in a luminescent working solution (WBKLS; Millipore, MA, USA) at room temperature for 3 min and scanned with an automatic chemiluminescence image analysis system (BIO-BEST; SIM, CA, USA). The information on all antibodies is displayed in Table 3, and GAPDH served as a loading control.

**Table 3:** All antibodies information and sources in Western blot in this study

ID	Catalog number	Company (country)	Molecular weight (kDa)	Dilution ratio
Bcl-2	#4223	CST (Massachusetts, USA)	26	1/1,000
Bax	#5023	CST (Massachusetts, USA)	20	1/1,000
Cleaved caspase-3	ab2302	Abcam (Cambridge, UK)	17	1/500
Caspase-3	ab32351	Abcam (Cambridge, UK)	35	1/5,000
GAPDH	ab181602	Abcam (Cambridge, UK)	36	1/10,000
Rabbit IgG	ab205718	Abcam (Cambridge, UK)		1/5,000

### 2.9 Statistical analysis

**Figure 1:**
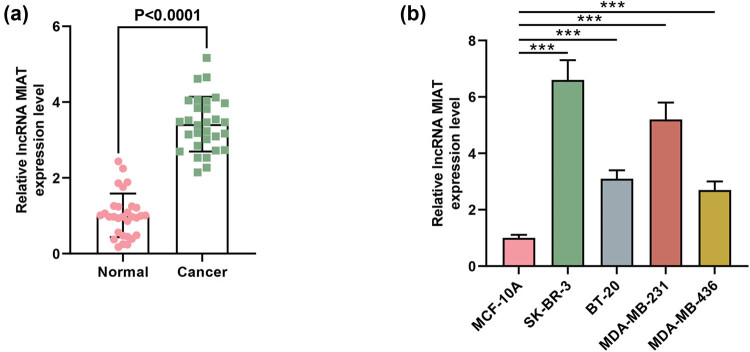
The expression of lncRNA MIAT was upregulated in breast cancer tissue samples and cell lines. (a) The expression of MIAT in breast cancer tissues was examined by qRT-PCR, and GAPDH was used as a reference gene. (b) The expression of MIAT in MCF-10A and breast cancer cell lines (SK-BR-3, BT-20, MDA-MB-231, and MDA-MB-436) was examined by qRT-PCR, and GAPDH was used as a reference gene. ^***^ *P* < 0.001.


In this study, measurement data were described by mean ± standard deviation. One-way analysis of variance was used for comparison among multiple groups, and the Tukey test was applied for pairwise comparison. In addition, a paired sample *t*-test was used for analyzing the data in Figures 1a and 5a, and an independent sample *t*-test was applied for comparison between the two groups in Table 1. All statistical analyses were implemented using Graphpad8.0 software and considered statistically significant at *P* < 0.05.

**Ethics statement:** All clinical operating procedures in this experiment were approved by the Ethics Committee of the Huai’an Second People’s Hospital (HEYLL202021). Written informed consent was obtained from all patients for the use of the collected specimens.

## 3 Results

### 3.1 MIAT was highly expressed in breast cancer, and high MIAT expression was associated with various clinical features of the patients

We determined the expression of MIAT in human breast cancer tissues and normal tissues by qRT-PCR. From Figure 1a, it can be observed that MIAT expression was markedly upregulated in tumor tissues as compared to that in the normal tissues. Similarly, qRT-PCR results also indicated that MIAT expression was increased in breast cancer cell lines when a comparison was made with that in the normal breast epithelial cell line MCF-10A (Figure 1b). In addition, we found that high MIAT expression was associated with various clinical characteristics of patients, including age, tumor size (V/cm^3^), low grade, ER status, HER2 status, P53 status, and tumor stage (Table 1).

### 3.2 siMIAT decreased breast cancer cell viability and proliferation and regulated the expression of apoptosis-related proteins

**Figure 2:**
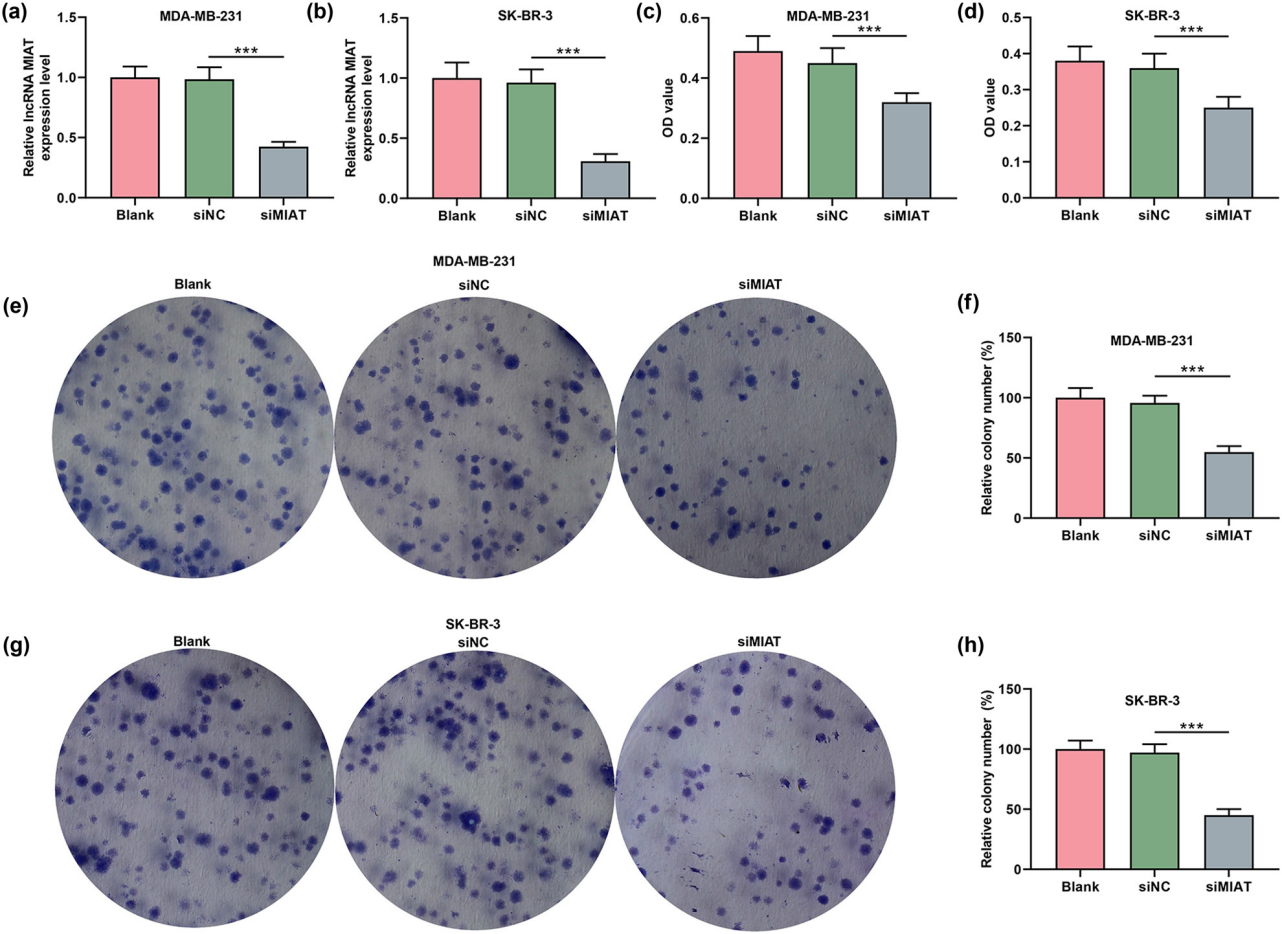
Silencing of MIAT decreased the viability and proliferation of breast cancer cells. (a and b) The expression of MIAT in MDA-MB-231 and SK-BR-3 cells after transfection with siMIAT was examined by qRT-PCR, and GAPDH was used as a reference gene. (c and d) The viability of MDA-MB-231 and SK-BR-3 cells after transfection with siMIAT was examined by the CCK-8 assay. (e–h) The proliferation abilities of MDA-MB-231 and SK-BR-3 cells after transfection with siMIAT were assessed by a colony formation assay. ^***^ *P* < 0.001. siMIAT: small interfering RNA targeting MIAT; siNC: negative control for siRNA.

**Figure 3:**
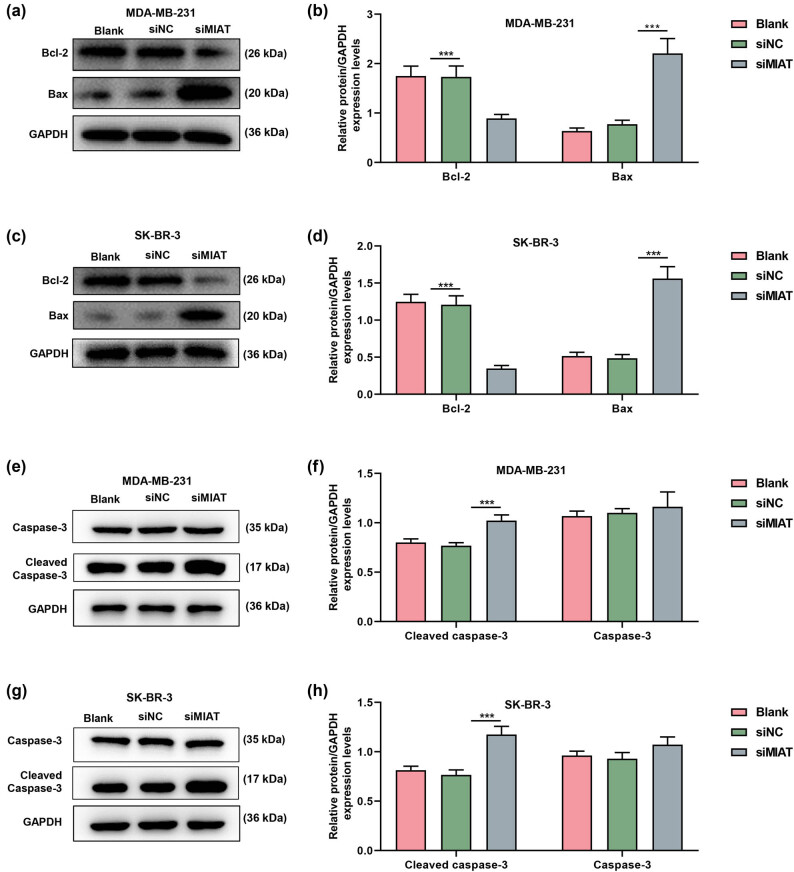
Silencing of MIAT regulated the expressions of apoptosis-related proteins in breast cancer cells. (a–d) The expressions of Bax and Bcl-2 in MDA-MB-231 and SK-BR-3 cells after transfection with siMIAT were examined by Western blot, and GAPDH was used as an internal loading control. (e–h) The expressions of caspase-3 and cleaved caspase-3 in MDA-MB-231 and SK-BR-3 cells after transfection with siMIAT were tested by Western blot, and GAPDH acted as an internal loading control. ^***^ *P* < 0.001. siMIAT: small interfering RNA targeting MIAT; siNC: negative control for siRNA.

As shown in Figure 2a and b, MIAT expression was downregulated by siMIAT in MDA-MB-231 and SK-BR-3 cells. Moreover, by testing the viability (Figure 2c and d) and proliferation (Figure 2e–h) of breast cancer cells after transfection, it can be proved that siMIAT inhibited the viability and proliferation compared to siNC. The expressions of apoptosis-related proteins in the transfected MDA-MB-231 (Figure 3a, b, e, and f) and SK-BR-3 cells (Figure 3c, d, g, and h) were also detected by Western blot. The data indicated that siMIAT elevated the expressions of cleaved caspase-3 and Bax while reducing Bcl-2 expression.

### 3.3 MIAT can sponge miR-378a-5p, and MIAT silencing promoted the expression of miR-378a-5p

**Figure 4:**
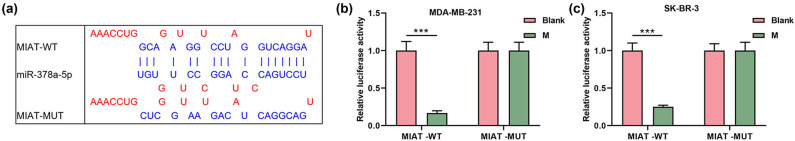
MIAT can sponge miR-378a-5p. (a) The binding relationship between MIAT and miR-378a-5p was predicted by DIANA tools-LncBase Experimental V2. (b and c) The binding relationship between MIAT and miR-378a-5p was validated by a dual-luciferase reporter assay. ^***^ *P* < 0.001. M: miR-378a-5p mimic; MUT: mutant type; WT: wild type.

**Figure 5:**
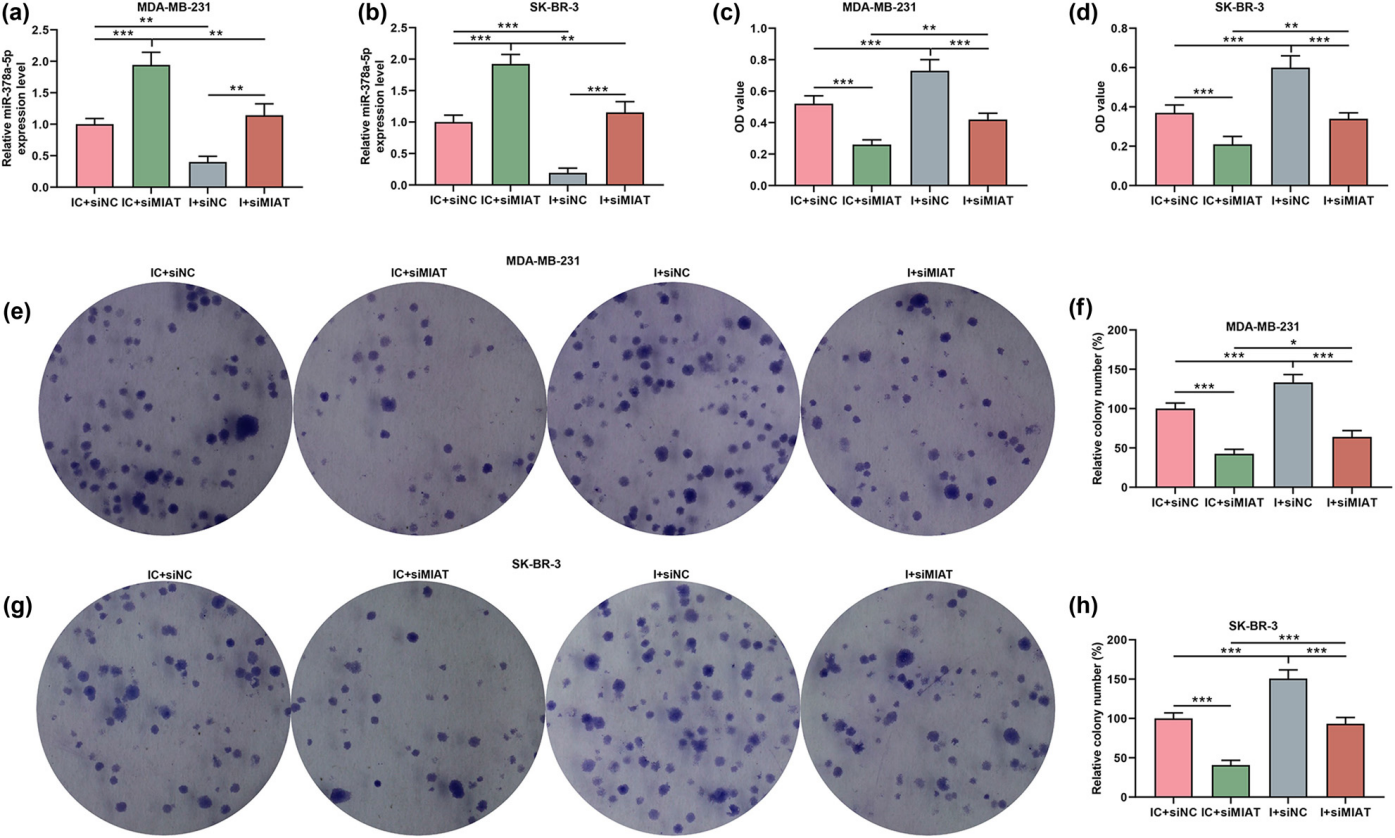
miR-378a-5p inhibitor counteracted the effects of siMIAT on breast cancer cell viability and proliferation. (a and b) The expression of miR-378a-5p in MDA-MB-231 and SK-BR-3 cells after transfection was examined by qRT-PCR, and U6 was applied as a reference gene. (c and d) The viability of MDA-MB-231 and SK-BR-3 cells after transfection was examined by the CCK-8 assay. (e–h) The proliferation abilities of MDA-MB-231 and SK-BR-3 cells after transfection were evaluated by a colony formation assay. ^*^ *P* < 0.05, ^**^ *P* < 0.01, ^***^ *P* < 0.001. I: miR-378a-5p inhibitor; IC: inhibitor control; siMIAT: small interfering RNA targeting MIAT; siNC: negative control for siRNA.

The binding sites of MIAT and miR-378a-5p were predicted by the DIANA tools-LncBase Experimental V2. As depicted in Figure 4a, there may be a binding relationship between MIAT and miR-378a-5p, which was subsequently validated using a dual-luciferase reporter assay. Based on Figure 4b and c, the luciferase activity was prominently reduced in breast cancer cells with co-transfection of miR-378a-5p mimic and MIAT-WT, as compared with that in cells with co-transfection of the mimic control and MIAT-WT. Besides, no distinct difference was observed in the breast cancer cells after co-transfection of miR-378a-5p mimic/mimic control and MIAT-MUT. It indicated that MIAT can sponge miR-378a-5p. In addition, miR-378a-5p expression in breast cancer cells was notably increased after transfection of siMIAT while being decreased after transfection of miR-378a-5p inhibitor, when comparison was made with that after transfection of IC and siNC (Figure 5a and b). Meanwhile, it can be observed that the miR-378a-5p inhibitor reversed the enhancing effect of siMIAT on miR-378a-5p expression in cells (Figure 5a and b).

### 3.4 miR-378a-5p inhibitor counteracted the effects of siMIAT on viability, proliferation, and the expressions of apoptosis-related proteins in breast cancer cells

**Figure 6:**
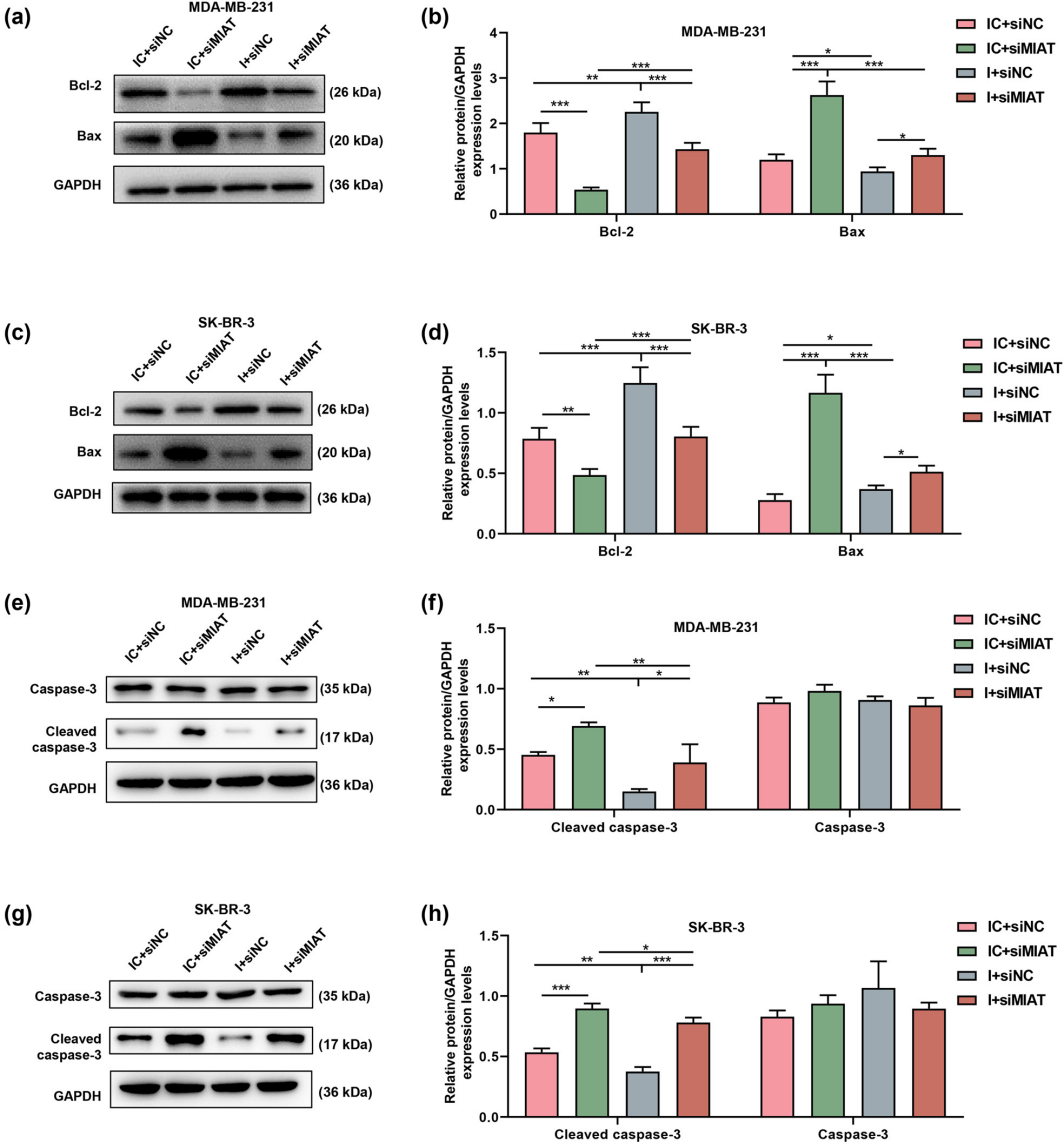
miR-378a-5p inhibitor reversed the effects of siMIAT on the expressions of apoptosis-related proteins in breast cancer cells. (a–d) The expressions of Bax, and Bcl-2 in MDA-MB-231 and SK-BR-3 cells after transfection were tested by Western blot, and GAPDH was utilized as an internal loading control. (e–h) The expressions of caspase-3 and cleaved caspase-3 in MDA-MB-231 and SK-BR-3 cells after transfection were determined by Western blot, and GAPDH was employed as an internal loading control. ^*^ *P* < 0.05, ^**^ *P* < 0.01, ^***^ *P* < 0.001. I: miR-378a-5p inhibitor; IC: inhibitor control; siMIAT: small interfering RNA targeting MIAT; siNC: negative control for siRNA.

The effects of miR-378a-5p on the breast cancer cells were detected. The results demonstrated that siMIAT suppressed but miR-378a-5p inhibitor promoted the breast cancer cell viability and proliferation, as compared with the IC and siNC (Figure 5c–h). Besides, the miR-378a-5p inhibitor offset the suppressive effect of siMIAT on cell viability and proliferation (Figure 5c–h). The experimental data from Western blot also illustrated that the miR-378a-5p inhibitor inhibited Bax and cleaved caspase-3 expressions while promoting Bcl-2 expression (Figure 6a–h). Similarly, miR-378a-5p inhibitors also neutralized the effect of siMIAT on these expressions of apoptosis-related proteins in breast cancer cells (Figure 6a–h).

### 3.5 miR-378a-5p expression was downregulated in breast cancer tissues and negatively correlated with MIAT expression

**Figure 7:**
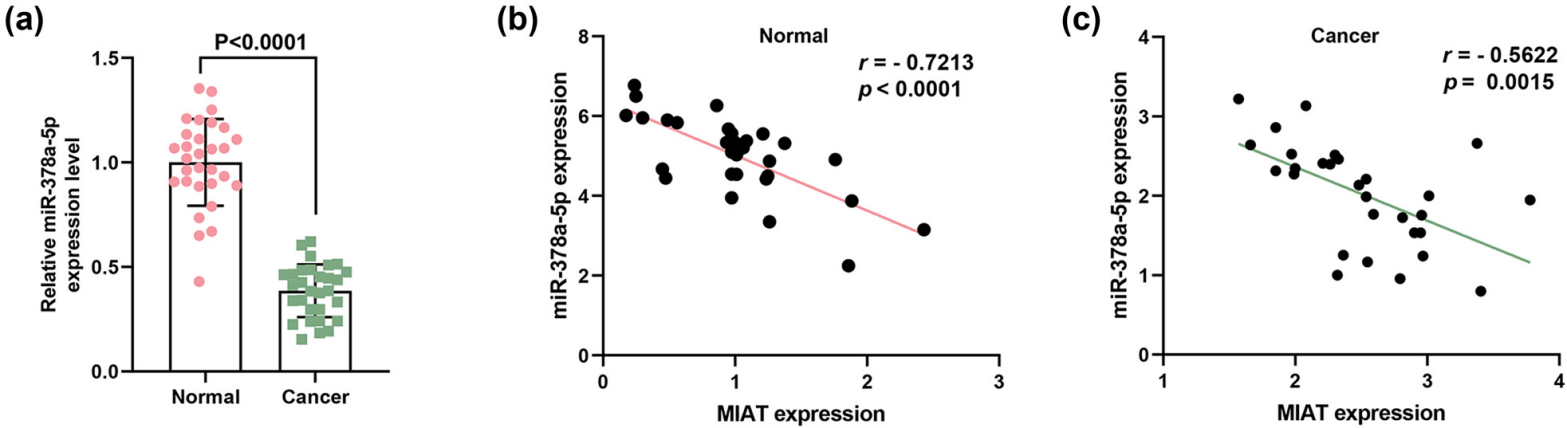
miR-378a-5p expression was downregulated in breast cancer tissues and negatively correlated with MIAT expression. (a) The expression of miR-378a-5p in breast cancer tissues was quantified by qRT-PCR, and U6 was used as a reference gene. (b and c) The expression relationship between MIAT and miR-378a-5p in breast cancer tissue samples and normal tissues was analyzed.

The expression of miR-378a-5p in breast cancer tissues was measured, and we discovered that miR-378a-5p expression was diminished in breast cancer tissues relative to that in human normal tissues (Figure 7a). After analyzing the relationship between miR-378a-5p and MIAT expressions in breast cancer tissues and normal tissues, we further found that their expressions were evidently negatively correlated (Figure 7b and c).

## 4 Discussion

At present, patients with breast cancer usually require surgical treatment to remove the tumor [26], and drugs or X-rays are also commonly used to destruct cancer cells in clinical practice [27,28]. However, the development mechanism of breast cancer is complex, and breast cancer has become one of the malignant tumors that are difficult to be cured due to its easy metastasis [29,30]. Substantial evidence has revealed that lncRNAs play key roles in tumor cell proliferation, apoptosis, etc., and their abnormal expressions have a certain relationship with tumor malignant grade, histological differentiation, and lymph node metastasis [9,31,32]. Thus, lncRNAs can act as potential biomarkers in the prognosis and diagnosis of assorted tumors [33].

MIAT has been proved to be highly expressed in breast cancer, and its aberrant expression is implicated in the clinical characteristics of patients with breast cancer [34]. Similarly, MIAT expression is upregulated in high-grade breast tumors, as well as ER- and Her2-positive tumor tissues [14]. Besides, MIAT expression is higher in ER-positive breast cancer tissues than in ER-negative tissues, and MIAT promotes estrogen-induced proliferation of ER-positive breast cancer cells [35]. In addition, MIAT is highly expressed in P53-negative cells (WTK1 cells) [36]. Consistent with what has been reported in previous studies, in this research, we found that the expression of MIAT was enhanced in breast cancer tissues and cells and that highly expressed MIAT was associated with multiple clinical characteristics of patients, including age, tumor size, low grade, ER status, HER2 status, P53 status, and tumor stage. These results indicate that MIAT can be applied to predict the degree of malignancy of breast tumors. Collectively, MIAT was found to be involved in regulating the development of breast cancer, but its specific role still needed further exploration.

Cancer-related death is mainly attributed to metastasis, and lncRNAs are involved in regulating a variety of physiological behaviors of cancer cells, including proliferation, migration, and apoptosis [37,38]. In gastric cancer, MIAT promotes the metastasis of cancer cells by mediating the expression of miR-141 [39]; in ovarian cancer, MIAT is involved in modulating the invasive process of cancer cells [40]; and in breast cancer cells, lncRNA TINCR and MIAT can regulate cell invasion, proliferation, and apoptosis [41,42]. Moreover, suppression of MIAT can cause G1 arrest in breast cancer cells, and MIAT may participate in tumorigenesis via regulation of the cell cycle [14,15,35]. Analogous to these findings, our experimental results uncovered that inhibition of MIAT reduced breast cancer cell viability and proliferation, but further verification still needed to be conducted at the molecular level.

Members of the Bcl-2 protein family play crucial roles in the process of apoptosis [43]. The Bcl-2 family can be divided into two major categories: one with anti-apoptotic effects (such as Bcl-2 and Bcl-W) and the other with pro-apoptotic effects (Bax, Bak, Bcl-XS, etc.) [43,44,45]. Caspases belong to the family of cysteine proteases and are key mediators of apoptosis [46]. Cleaved caspase-3 is an activated form of caspase-3, and high expression of cleaved caspase-3 in cells promotes apoptosis [47]. Our experiments demonstrated that siMIAT may promote the apoptosis of breast cancer cells by regulating the expressions of apoptosis-related proteins. These results provided a mechanistic basis to fathom the interaction between MIAT and miR-378a-5p in breast cancer.

As previously documented, miR-378a-5p impacts the physiological behaviors of oral squamous cell carcinoma cells and colorectal cancer cells, such as migration, angiogenesis, and apoptosis [19,20], and its expression was low in both colorectal and breast cancer cells. In triple-negative breast cancer cells, lncRNA GAS5 promotes cancer cell apoptosis by targeting miR-378a-5p, and the targeting relationship of miR-378a-5p and cyclin G2 has been confirmed by luciferase reporter assay in BeWo cells [18,20,21,48]. On this basis, this research further unveiled that miR-378a-5p expression was regulated by MIAT and that miR-378a-5p inhibitor countervailed the effects of siMIAT on the viability, proliferation, and expressions of apoptosis-related proteins in breast cancer cells. It can be concluded that MIAT silencing inhibits the viability and proliferation of breast cancer cells by promoting the expression of miR-378a-5p.

In conclusion, this research confirms that MIAT is highly expressed but miR-378a-5p expression is low in breast cancer cells, and MIAT silencing inhibits the viability and proliferation of breast cancer cells by promoting the expression of miR-378a-5p. These results, to some extent, unveil an underlying molecular mechanism of breast cancer development and provide potential new targets for the treatment of breast cancer.

## References

[1] Manning L, Holmes J, Bonin K, Vidi PA. Radial profile analysis of epithelial polarity in breast acini: a tool for primary (breast) cancer prevention. Front Med (Lausanne). 2019;6:314. 10.3389/fmed.2019.00314 PMC6970192 31998733

[2] Li X, Zeng Z, Wang J, Wu Y, Chen W, Zheng L, et al. MicroRNA-9 and breast cancer. Biomed Pharmacother. 2020;122:109687. 10.1016/j.biopha.2019.109687 31918267

[3] Milosevic M, Jankovic D, Milenkovic A, Stojanov D. Early diagnosis and detection of breast cancer. Technol Health Care. 2018;26(4):729–59. 10.3233/THC-181277 30124455

[4] Leon-Ferre RA, Majithia N, Loprinzi CL. Management of hot flashes in women with breast cancer receiving ovarian function suppression. Cancer Treat Rev. 2017;52:82–90. 10.1016/j.ctrv.2016.11.012 27960127

[5] Manca G, Tardelli E, Rubello D, Gennaro M, Marzola MC, Cook GJ, et al. Sentinel lymph node biopsy in breast cancer: a technical and clinical appraisal. Nucl Med Commun. 2016;37(6):570–6. 10.1097/MNM.0000000000000489 26886421

[6] Engel J, Weichert W, Jung A, Emeny R, Holzel D. Lymph node infiltration, parallel metastasis and treatment success in breast cancer. Breast. 2019;48:1–6. 10.1016/j.breast.2019.07.008 31415842

[7] Nagini S. Breast cancer: current molecular therapeutic targets and new players. Anticancer Agents Med Chem. 2017;17(2):152–63. 10.2174/1871520616666160502122724 27137076

[8] Li J, Jiang X, Li Z, Huang L, Zhou Y, Liu Y, et al. Long noncoding RNA GHET1 in human cancer. Clin Chim Acta. 2019;488:111–5. 10.1016/j.cca.2018.11.007 30399371

[9] Bhan A, Soleimani M, Mandal SS. Long noncoding RNA and cancer: a new paradigm. Cancer Res. 2017;77(15):3965–81. 10.1158/0008-5472.CAN-16-2634 PMC8330958 28701486

[10] Ishii N, Ozaki K, Sato H, Mizuno H, Susumu S, Takahashi A, et al. Identification of a novel non-coding RNA, MIAT, that confers risk of myocardial infarction. J Hum Genet. 2006;51(12):1087–99. 10.1007/s10038-006-0070-9 17066261

[11] Liu W, Wang Z, Wang C, Ai Z. Long non-coding RNA MIAT promotes papillary thyroid cancer progression through upregulating LASP1. Cancer Cell Int. 2019;19:194. 10.1186/s12935-019-0913-z PMC6659215 31372094

[12] Li DS, Ainiwaer JL, Sheyhiding I, Zhang Z, Zhang LW. Identification of key long non-coding RNAs as competing endogenous RNAs for miRNA-mRNA in lung adenocarcinoma. Eur Rev Med Pharmacol Sci. 2016;20(11):2285–95. 27338053

[13] Crea F, Venalainen E, Ci X, Cheng H, Pikor L, Parolia A, et al. The role of epigenetics and long noncoding RNA MIAT in neuroendocrine prostate cancer. Epigenomics. 2016;8(5):721–31. 10.2217/epi.16.6 27096814

[14] Alipoor FJ, Asadi MH, Torkzadeh-Mahani M. MIAT lncRNA is overexpressed in breast cancer and its inhibition triggers senescence and G1 arrest in MCF7 cell line. J Cell Biochem. 2018;119(8):6470–81. 10.1002/jcb.26678 29345338

[15] Almnaseer ZA, Mourtada-Maarabouni M. Long noncoding RNA MIAT regulates apoptosis and the apoptotic response to chemotherapeutic agents in breast cancer cell lines. Biosci Rep. 2018;38(4):BSR20180704. 10.1042/BSR20180704 PMC6435567 29914974

[16] Zhao W, Geng D, Li S, Chen Z, Sun M. LncRNA HOTAIR influences cell growth, migration, invasion, and apoptosis via the miR-20a-5p/HMGA2 axis in breast cancer. Cancer Med. 2018;7(3):842–55. 10.1002/cam4.1353 PMC5852357 29473328

[17] Zhen Q, Gao LN, Wang RF, Chu WW, Zhang YX, Zhao XJ, et al. LncRNA DANCR promotes lung cancer by sequestering miR-216a. Cancer Control. 2018;25(1):1073274818769849. 10.1177/1073274818769849 PMC6852365 29651883

[18] Zheng S, Li M, Miao K, Xu H. lncRNA GAS5-promoted apoptosis in triple-negative breast cancer by targeting miR-378a-5p/SUFU signaling. J Cell Biochem. 2020;121(3):2225–35. 10.1002/jcb.29445 31692053

[19] Cui Z, Liu QL, Sun SQ, Jiao K, Liu DR, Zhou XC, et al. MiR-378a-5p inhibits angiogenesis of oral squamous cell carcinoma by targeting KLK4. Neoplasma. 2020;67(1):85–92. 10.4149/neo_2019_190306N191 31829025

[20] Li H, Dai S, Zhen T, Shi H, Zhang F, Yang Y, et al. Clinical and biological significance of miR-378a-3p and miR-378a-5p in colorectal cancer. Eur J Cancer. 2014;50(6):1207–21. 10.1016/j.ejca.2013.12.010 24412052

[21] Winsel S, Maki-Jouppila J, Tambe M, Aure MR, Pruikkonen S, Salmela AL, et al. Excess of miRNA-378a-5p perturbs mitotic fidelity and correlates with breast cancer tumourigenesis in vivo. Br J Cancer. 2014;111(11):2142–51. 10.1038/bjc.2014.524 PMC4260036 25268374

[22] Shen Y, Dong LF, Zhou RM, Yao J, Song YC, Yang H, et al. Role of long non-coding RNA MIAT in proliferation, apoptosis and migration of lens epithelial cells: a clinical and in vitro study. J Cell Mol Med. 2016;20(3):537–48. 10.1111/jcmm.12755 PMC4759467 26818536

[23] Goldar S, Khaniani MS, Derakhshan SM, Baradaran B. Molecular mechanisms of apoptosis and roles in cancer development and treatment. Asian Pac J Cancer Prev. 2015;16(6):2129–44. 10.7314/apjcp.2015.16.6.2129 25824729

[24] Wong RS. Apoptosis in cancer: from pathogenesis to treatment. J Exp Clin Cancer Res. 2011;30(1):87. 10.1186/1756-9966-30-87 PMC3197541 21943236

[25] Warren CFA, Wong-Brown MW, Bowden NA. BCL-2 family isoforms in apoptosis and cancer. Cell Death Dis. 2019;10(3):177. 10.1038/s41419-019-1407-6 PMC6384907 30792387

[26] Jonczyk MM, Jean J, Graham R, Chatterjee A. Surgical trends in breast cancer: a rise in novel operative treatment options over a 12 year analysis. Breast Cancer Res Treat. 2019;173(2):267–74. 10.1007/s10549-018-5018-1 PMC6486837 30361873

[27] Arslan C, Altundag K, Dizdar O. Emerging drugs in metastatic breast cancer: an update. Expert Opin Emerg Drugs. 2011;16(4):647–67. 10.1517/14728214.2011.640672 22122529

[28] Merino Bonilla JA, Torres Tabanera M, Ros Mendoza LH. Breast cancer in the 21st century: from early detection to new therapies. Radiologia. 2017;59(5):368–79. 10.1016/j.rx.2017.06.003 28712528

[29] Wen C, Wu L, Fu L, Wang B, Zhou H. Unifying mechanism in the initiation of breast cancer by metabolism of estrogen (Review). Mol Med Rep. 2017;16(2):1001–6. 10.3892/mmr.2017.6738 28627646

[30] Scully OJ, Bay BH, Yip G, Yu Y. Breast cancer metastasis. Cancer Genomics Proteom. 2012;9(5):311–20. 22990110

[31] Lim LJ, Wong SYS, Huang F, Lim S, Chong SS, Ooi LL, et al. Roles and regulation of long noncoding RNAs in hepatocellular carcinoma. Cancer Res. 2019;79(20):5131–9. 10.1158/0008-5472.CAN-19-0255 31337653

[32] Ji D, Zhong X, Jiang X, Leng K, Xu Y, Li Z, et al. The role of long non-coding RNA AFAP1-AS1 in human malignant tumors. Pathol Res Pract. 2018;214(10):1524–31. 10.1016/j.prp.2018.08.014 30173945

[33] Fan CN, Ma L, Liu N. Systematic analysis of lncRNA-miRNA-mRNA competing endogenous RNA network identifies four-lncRNA signature as a prognostic biomarker for breast cancer. J Transl Med. 2018;16(1):264. 10.1186/s12967-018-1640-2 PMC6161429 30261893

[34] Tang J, Ren J, Cui Q, Zhang D, Kong D, Liao X, et al. A prognostic 10-lncRNA expression signature for predicting the risk of tumour recurrence in breast cancer patients. J Cell Mol Med. 2019;23(10):6775–84. 10.1111/jcmm.14556 PMC6787455 31429520

[35] Li Y, Jiang B, Wu X, Huang Q, Chen W, Zhu H, et al. Long non-coding RNA MIAT is estrogen-responsive and promotes estrogen-induced proliferation in ER-positive breast cancer cells. Biochem Biophys Res Commun. 2018;503(1):45–50. 10.1016/j.bbrc.2018.05.146 29792859

[36] Chaudhry MA. Expression pattern of small nucleolar RNA host genes and long non-coding RNA in X-rays-treated lymphoblastoid cells. Int J Mol Sci. 2013;14(5):9099–110. 10.3390/ijms14059099 PMC3676775 23698766

[37] Gilkes DM, Semenza GL, Wirtz D. Hypoxia and the extracellular matrix: drivers of tumour metastasis. Nat Rev Cancer. 2014;14(6):430–9. 10.1038/nrc3726 PMC4283800 24827502

[38] Wang AH, Fan WJ, Fu L, Wang XT. LncRNA PCAT-1 regulated cell proliferation, invasion, migration and apoptosis in colorectal cancer through targeting miR-149-5p. Eur Rev Med Pharmacol Sci. 2019;23(19):8310–20. 10.26355/eurrev_201910_19142 31646561

[39] Sha M, Lin M, Wang J, Ye J, Xu J, Xu N, et al. Long non-coding RNA MIAT promotes gastric cancer growth and metastasis through regulation of miR-141/DDX5 pathway. J Exp Clin Cancer Res. 2018;37(1):58. 10.1186/s13046-018-0725-3 PMC5852965 29540201

[40] Zhou S, Xu A, Song T, Gao F, Sun H, Kong X. lncRNA MIAT regulates cell growth, migration, and invasion through sponging miR-150-5p in ovarian cancer. Cancer Biother Radiopharm. 2020;35(9):650–60. 10.1089/cbr.2019.3259 32186927

[41] Guo F, Zhu X, Zhao Q, Huang Q. miR5893p sponged by the lncRNA TINCR inhibits the proliferation, migration and invasion and promotes the apoptosis of breast cancer cells by suppressing the Akt pathway via IGF1R. Int J Mol Med. 2020;46(3):989–1002. 10.3892/ijmm.2020.4666 PMC7388824 32705168

[42] Luan T, Zhang X, Wang S, Song Y, Zhou S, Lin J, et al. Long non-coding RNA MIAT promotes breast cancer progression and functions as ceRNA to regulate DUSP7 expression by sponging miR-155-5p. Oncotarget. 2017;8(44):76153–64. 10.18632/oncotarget.19190 PMC5652694 29100300

[43] Dietrich JB. Apoptosis and anti-apoptosis genes in the Bcl-2 family. Arch Physiol Biochem. 1997;105(2):125–35. 10.1076/apab.105.2.125.12927 9296841

[44] Tse C, Shoemaker AR, Adickes J, Anderson MG, Chen J, Jin S, et al. ABT-263: a potent and orally bioavailable Bcl-2 family inhibitor. Cancer Res. 2008;68(9):3421–8. 10.1158/0008-5472.CAN-07-5836 18451170

[45] Moldoveanu T, Czabotar PE. BAX, BAK, and BOK: a coming of age for the BCL-2 family effector proteins. Cold Spring Harb Perspect Biol. 2020;12(4):a036319. 10.1101/cshperspect.a036319 PMC7111251 31570337

[46] Chowdhury I, Tharakan B, Bhat GK. Current concepts in apoptosis: the physiological suicide program revisited. Cell Mol Biol Lett. 2006;11(4):506–25. 10.2478/s11658-006-0041-3 PMC6275981 16977376

[47] Kavanagh E, Rodhe J, Burguillos MA, Venero JL, Joseph B. Regulation of caspase-3 processing by cIAP2 controls the switch between pro-inflammatory activation and cell death in microglia. Cell Death Dis. 2014;5:e1565. 10.1038/cddis.2014.514 PMC4454160 25501826

[48] Nadeem U, Ye G, Salem M, Peng C. MicroRNA-378a-5p targets cyclin G2 to inhibit fusion and differentiation in BeWo cells. Biol Reprod. 2014;91(3):76. 10.1095/biolreprod.114.119065 25122062

